# Supplementary material for: Highly active and selective oxygen reduction to H2O2 on boron-doped carbon for high production rates
Source: Nat Commun. 2021 Jul 9;12:4225. doi: 10.1038/s41467-021-24329-9 (PMC8270976; doi:10.1038/s41467-021-24329-9)
Supplement: Supplementary file 1 — Supplementary Information [file 41467_2021_24329_MOESM1_ESM.pdf]

Supporting Information for

Highly Active and Selective Oxygen Reduction to H_2O_2 on Boron-Doped Carbon for High Production Rates

Yang Xia[1,†], Xunhua Zhao[2,†], Chuan Xia[1,3], Zhen-Yu Wu[1], Peng Zhu[1], Jung Yoon (Timothy) Kim[1], Xiaowan Bai[2], Guanhui Gao[4], Yongfeng Hu[5], Jun Zhong[6], Yuanyue Liu[2]*, Haotian Wang[1,3,7]*

*Correspondence:

yuanyue.liu@austin.utexas.edu (Y. Liu)

htwang@rice.edu (H. Wang)

[†] These authors contributed equally.

This PDF file includes:

Supplementary Figures. 1 to 33

Supplementary Tables. 1 to 8

Supplementary Note 1

Supplementary References

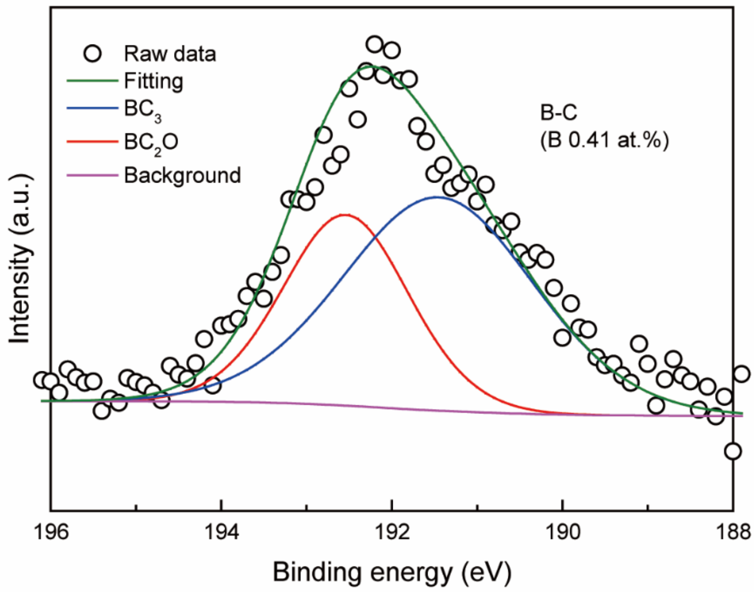

Supplementary Figure 1. High resolution boron XPS peak analysis of B-C. The trace amount of oxygen species remained comes from the adsorption of water and air during XPS sample preparation and transportation, and possible trace of remnant oxygen species from high temperature annealing (see more details in **Supplementary Figure 15 and 16**). Note that a.u. represents arbitrary units.

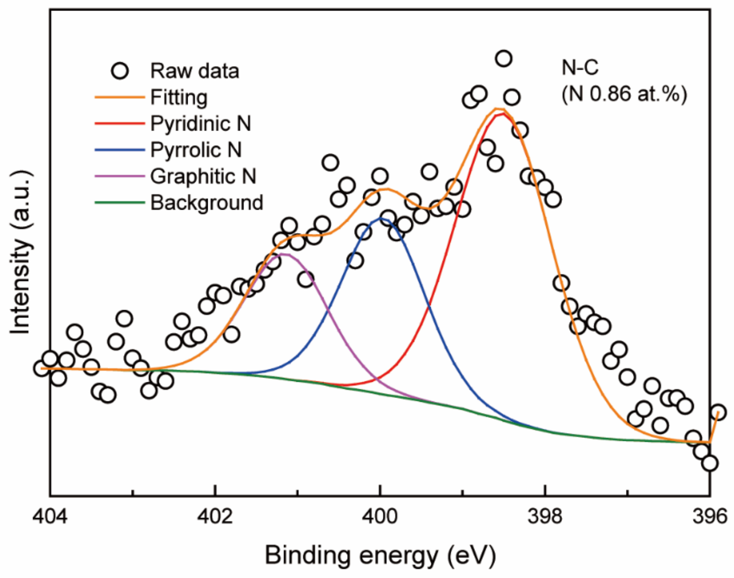

Supplementary Figure 2. High resolution nitrogen XPS peak analysis of N-C. Note that a.u. represents arbitrary units.

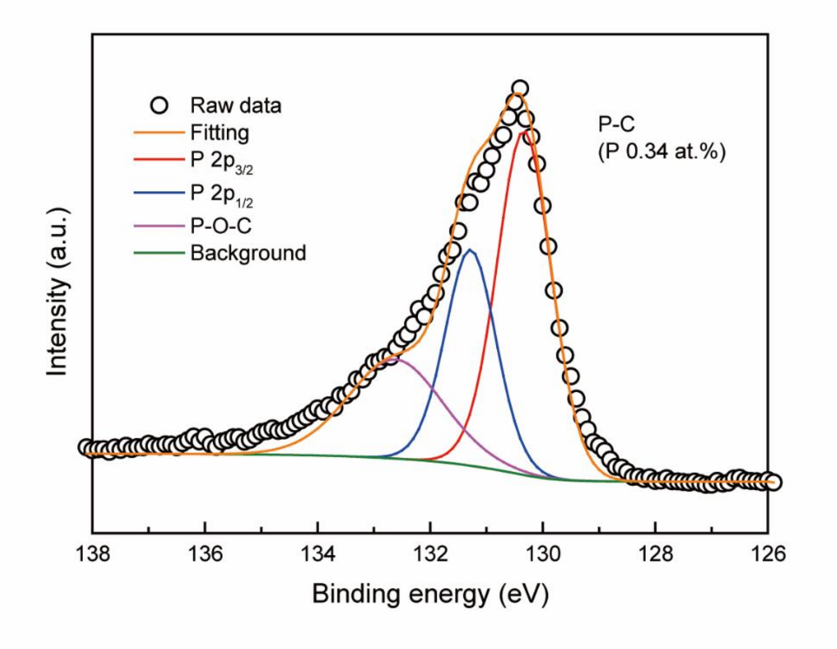

Supplementary Figure 3. High resolution phosphorous XPS peak analysis of P-C. The trace amount of oxygen species remained comes from the adsorption of water and air during XPS sample preparation and transportation (see more details in **Supplementary Figure 15**). Note that a.u. represents arbitrary units.

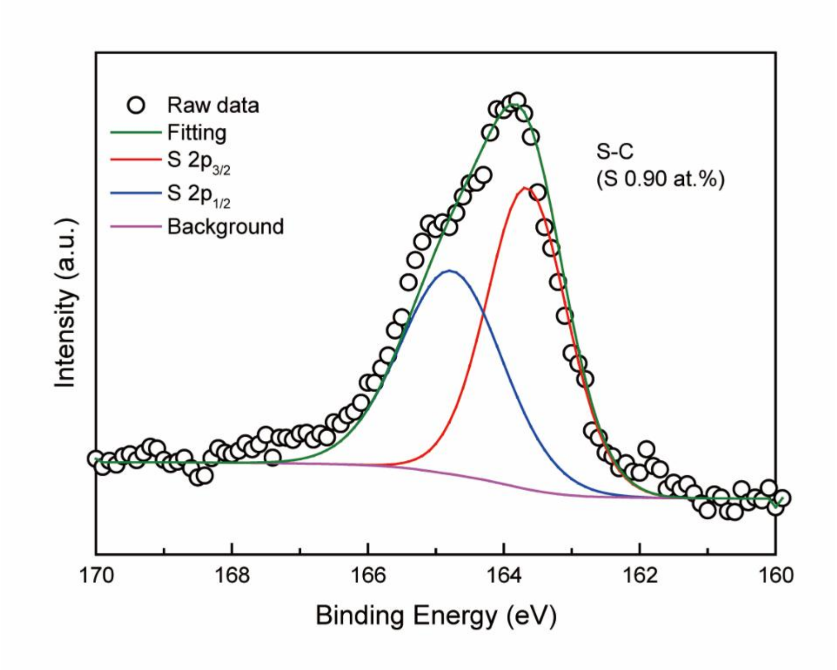

Supplementary Figure 4. High resolution sulfur XPS peak analysis of S-C. Note that a.u. represents arbitrary units.

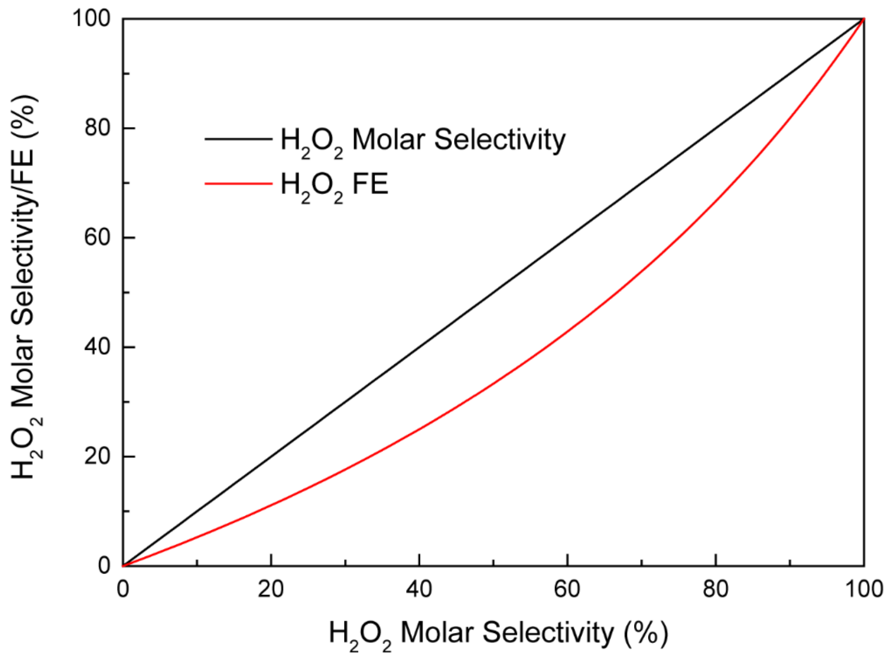

Supplementary Figure 5. Correlation between H_2O_2 molar selectivity and H_2O_2 Faradaic efficiency (FE) in Rotating Ring-Disk Electrode (RRDE) measurement. Note that the increase in FE does not obey the same linear relationship as that of molar selectivity.

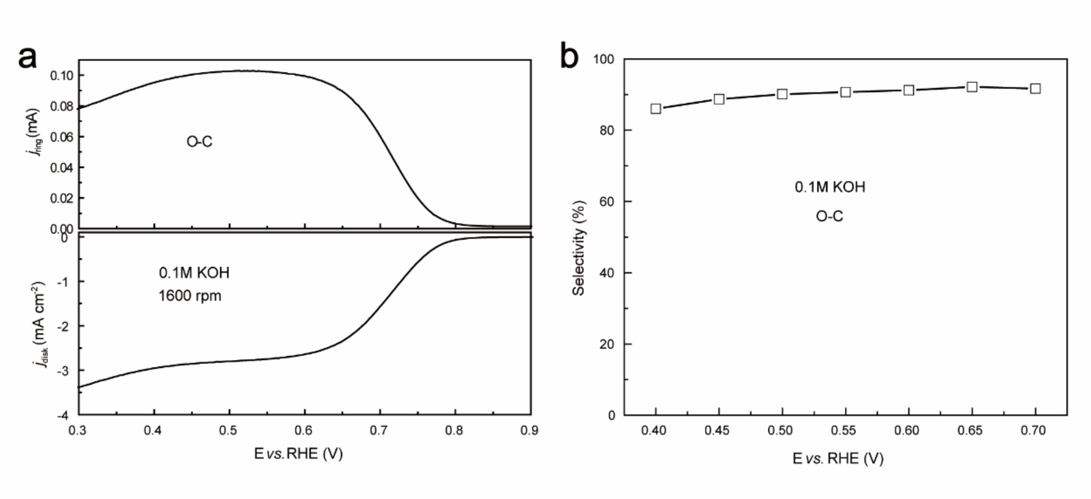

Supplementary Figure 6. ORR performance of O-C by RRDE 0.1M KOH. (a) Linear sweep voltammetry (LSV) (b) H_2O_2 molar selectivity.

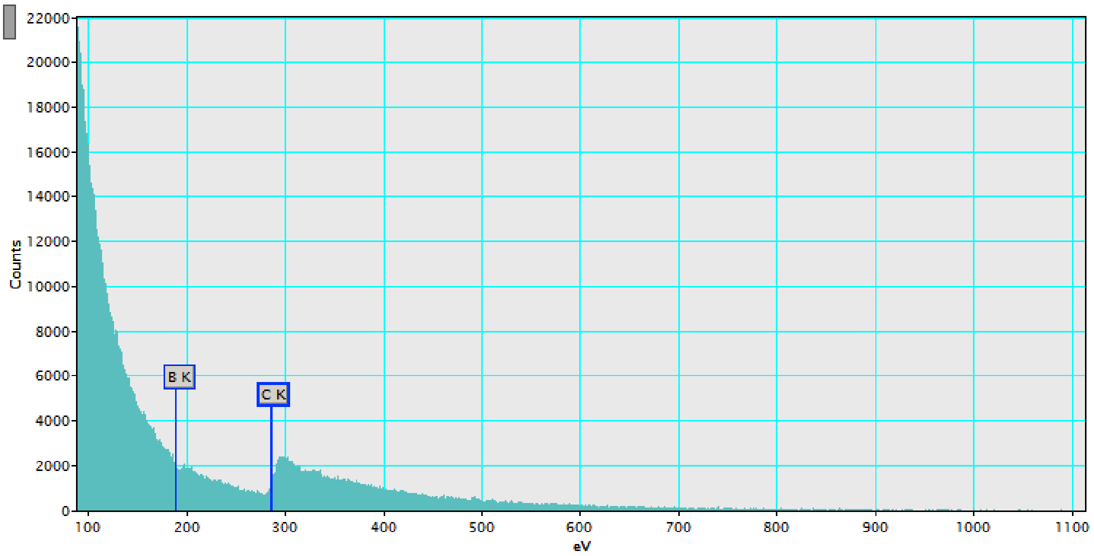

Supplementary Figure 7. Extracted EELS spectrum of B-C using TEM. B K-edge peak and C K-edge peak are shown on the graph, respectively. The EELS spectrum confirms the successful doping of boron on the carbon black substrate.

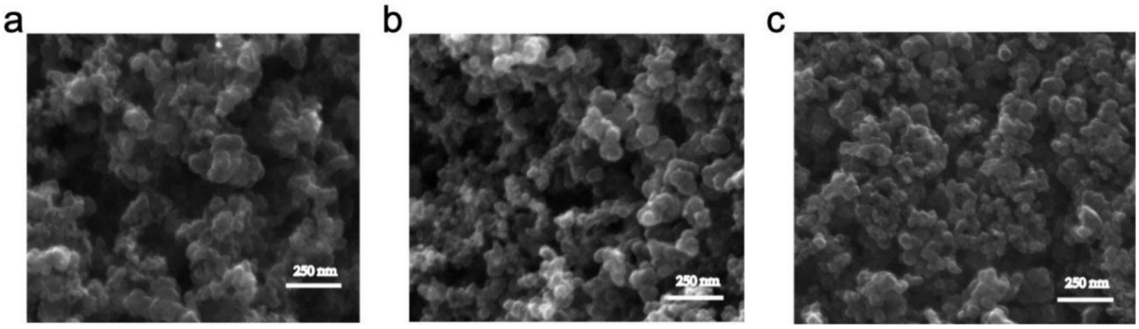

Supplementary Figure 8. SEM image for morphology of (a) N-C **(b)** P-C **(c)** S-C**.** The scale bars represent 250 nm length. The intact carbon black morphology shown here in SEM of all the samples excludes any morphological effect on the variation of $2e^-$-ORR performance in RRDE.

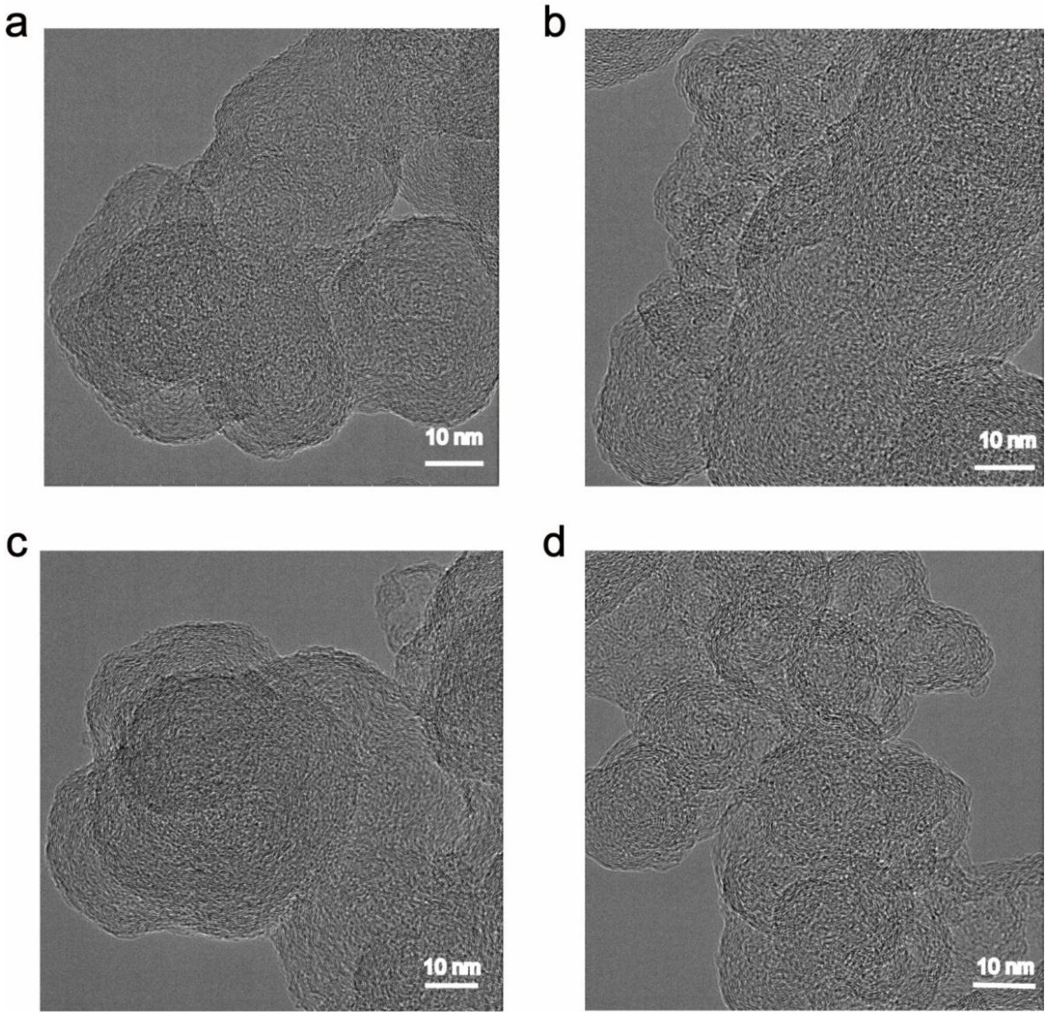

Supplementary Figure 9. High-resolution TEM image for (a) B-C **(b)** N-C **(c)** P-C **(d)** S-C**.** The scale bars represent 10 nm length. The morphology is similar among all different doped catalysts, and there is no nano-scale difference in surface roughness.

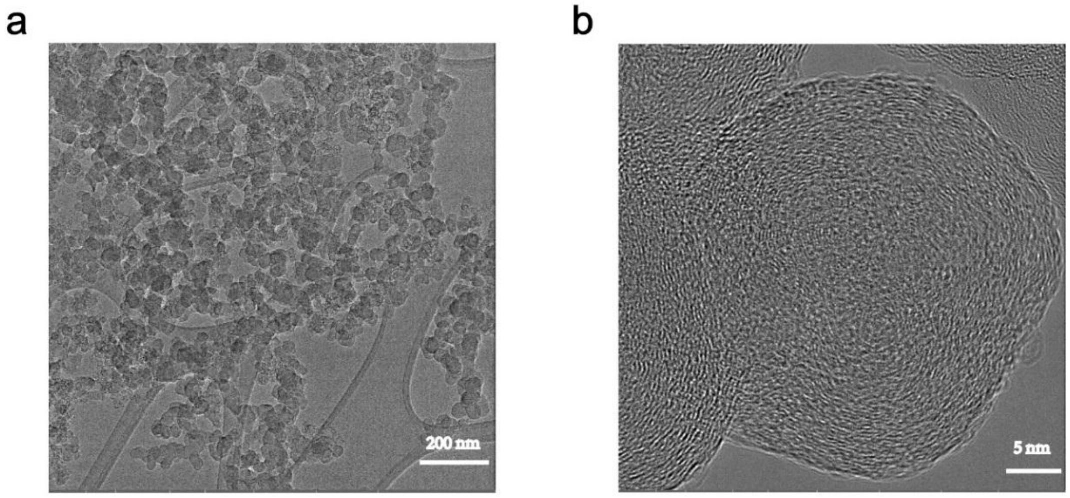

Supplementary Figure 10. High-resolution TEM image for B-C under (a) 200 nm **(b)** 5 nm**.**

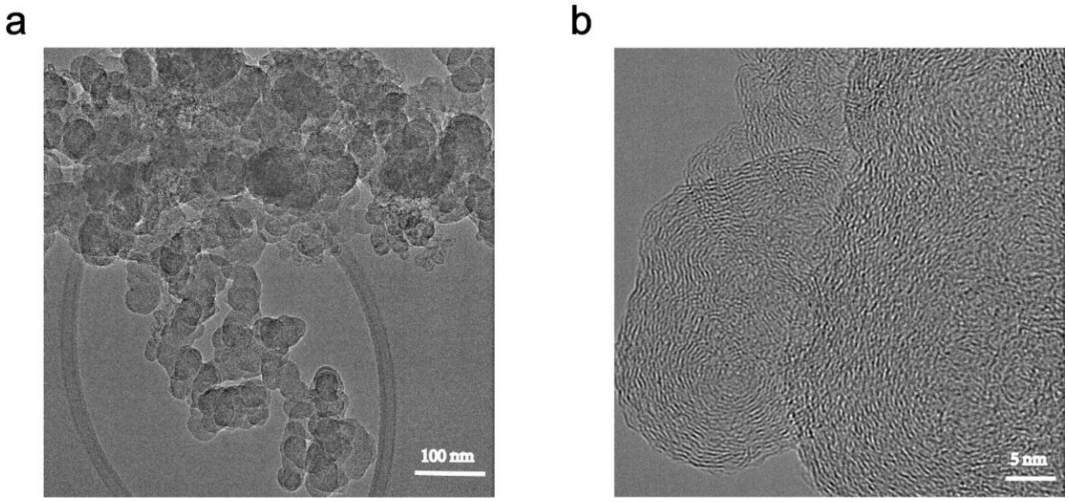

Supplementary Figure 11. High-resolution TEM image for N-C under (a) 100 nm **(b)** 5 nm**.**

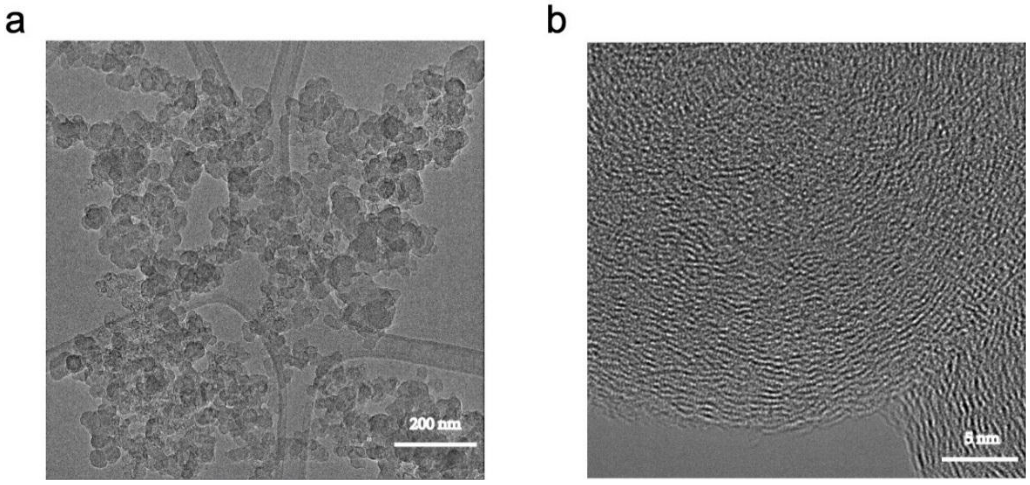

Supplementary Figure 12. High-resolution TEM image for P-C under (a) 200 nm **(b)** 5 nm**.**

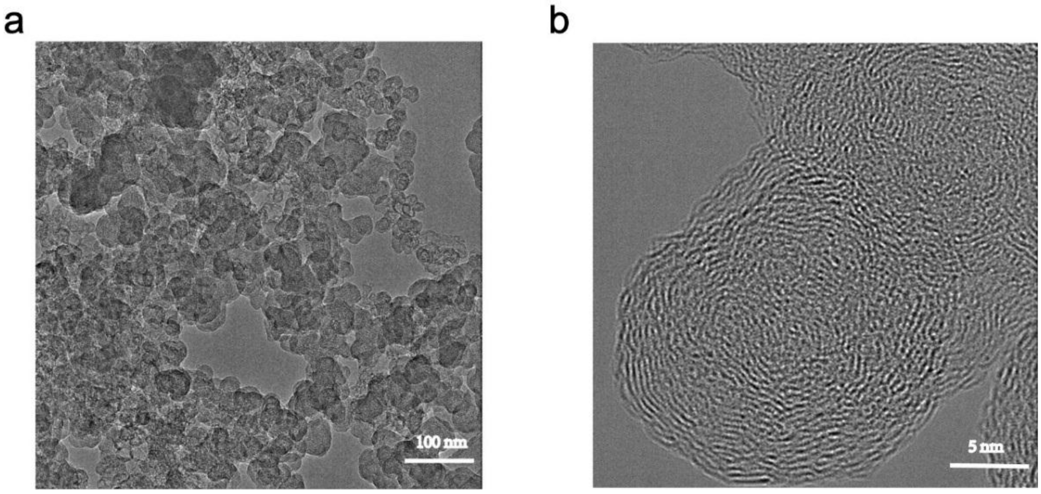

Supplementary Figure 13. High-resolution TEM image for S-C under (a) 100 nm **(b)** 5 nm**.**

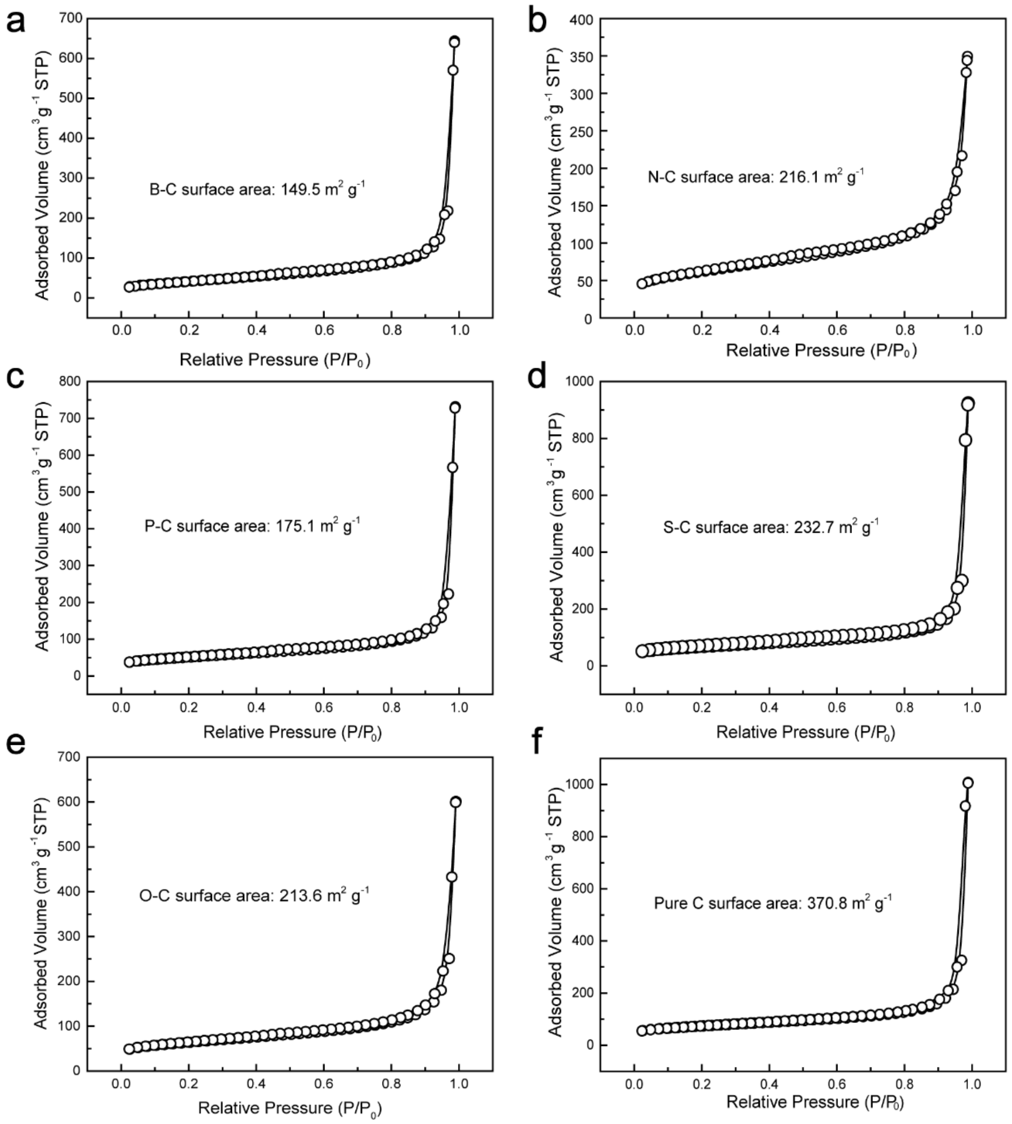

Supplementary Figure 14. BET surface area analysis for (a) B-C (b) N-C (c) P-C (d) S-C (e) O-C (f) Pure C. Note that all the BET surface areas were measured similar or slightly higher compared to B-C, excluding any possible contribution of the surface area increase to enhanced H_2O_2 performance of B-C.

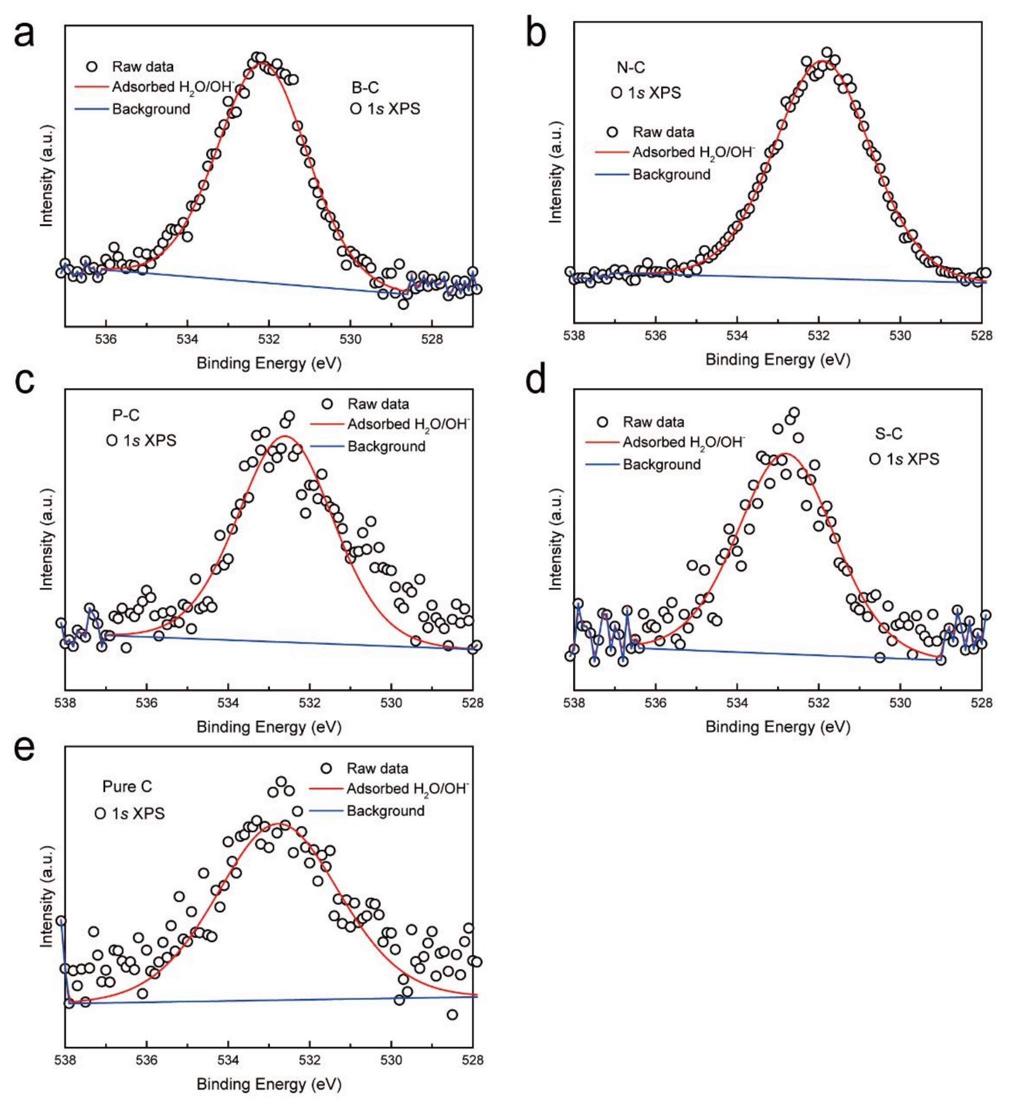

Supplementary Figure 15. High resolution oxygen XPS peak analysis of (a) B-C (b) N-C (c) P-C (d) S-C (e) Pure C, respectively. The peak located at 532~533eV for all the five samples corresponds to surface adsorbed H_2O/OH^- species. Since there is no peak of lattice oxygen (around 529~530eV)[1] observed, we can conclude that the trace amount of oxygen species remained come from the adsorption of water and air during XPS sample preparation and transportation. Note that a.u. represents arbitrary units.

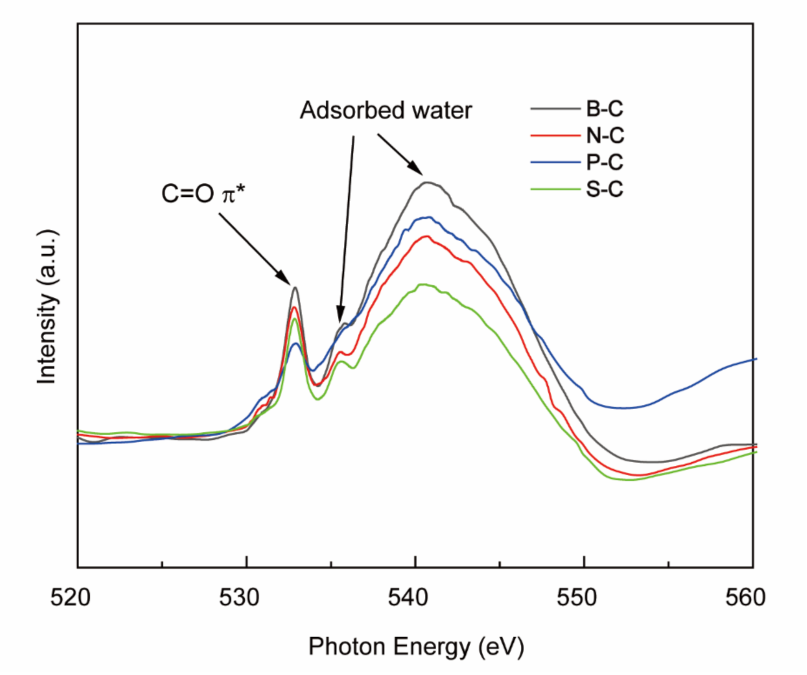

Supplementary Figure 16. NEXAFS O K-edge spectra of B-C, N-C, P-C and S-C. The peak located at ~533 eV for all the samples corresponds to π-bonded oxygen on carbon surface either from trace amount of oxygen dopant which is impossible to remove when annealing the samples at the high temperature (750 ^{o}C) or due to the oxidation during the sample exposure to air. The peaks around 535 eV and 540 eV are assigned to surface adsorbed/residual water species, which is consistent with our XPS results shown in Supplementary Figure 15. Note that a.u. represents arbitrary units.

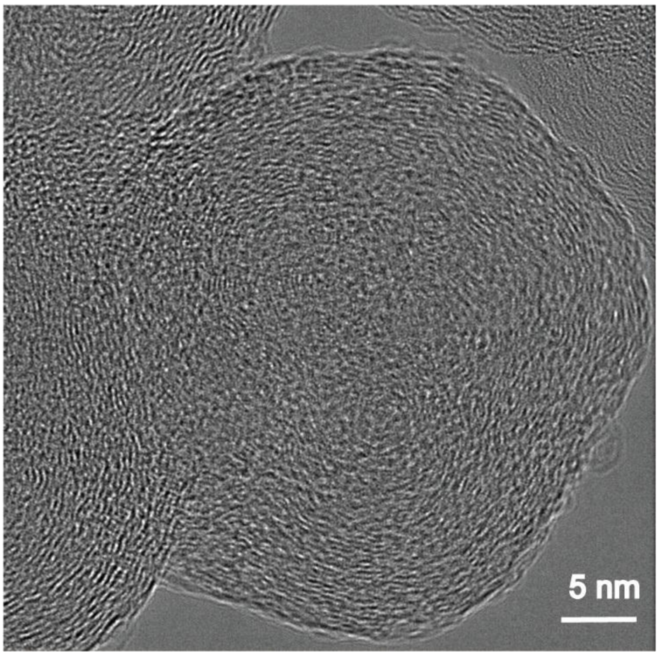

Supplementary Figure 17. High resolution TEM image of CB. Note that the local structure in nano-meter scale is exactly graphene-like structure. Actually, the structure of carbon black is comparable to graphite: both are composed of graphene sheets, while graphite's layers are typically larger and more ordered than carbon black whose sheets form 3-dimensional structure. Based on this information, graphene structure is typically used to represent carbon black for modelling electrochemistry[2,3]. Considering the 3-dimensional structure of carbon black would be too large for DFT modeling while chemical reaction typically takes place at ~1 nm scale, we believe using graphene as the carbon structure in our simulations is appropriate to our best computational resources.

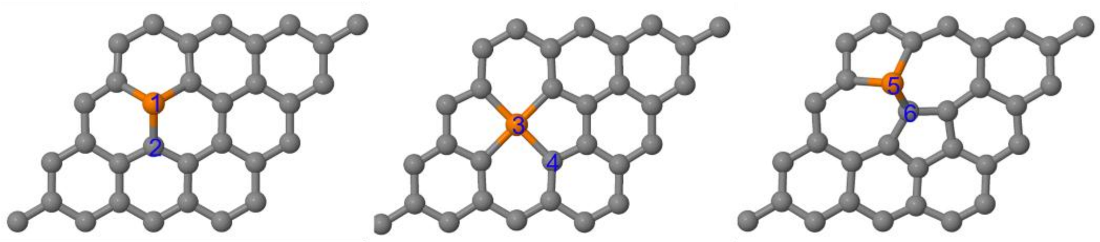

Supplementary Figure 18. Illustration of six adsorption sites that considered in this work. The orange sphere represents the dopant atom while the grey spheres are the carbon atoms. 1,2,3,4,5,6 sites are named as “SV”, “C-SV”, “DV”, “C-DV”, “5577” and “C-5577” respectively.

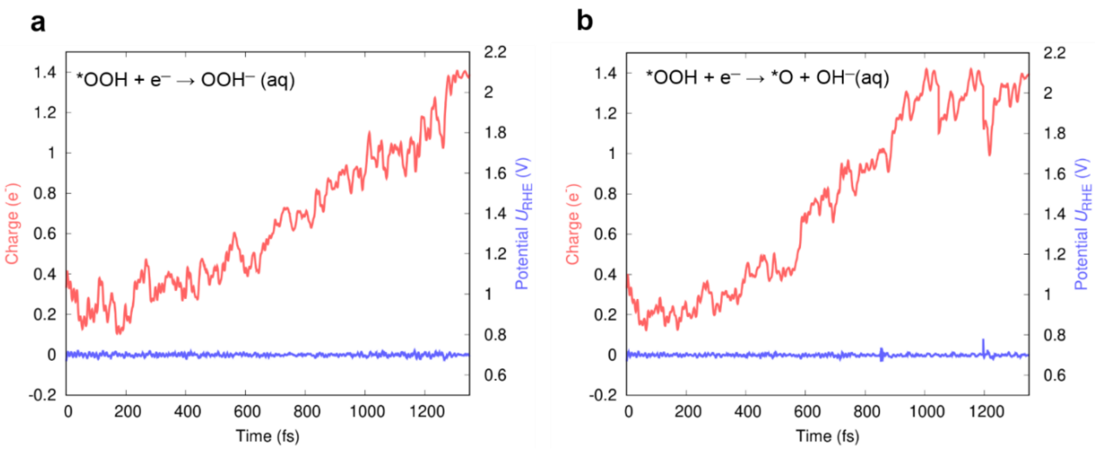

Supplementary Figure 19. The evolution of net charge in charge transfer at fixed constant potential of 0.7 V *vs.* RHE.

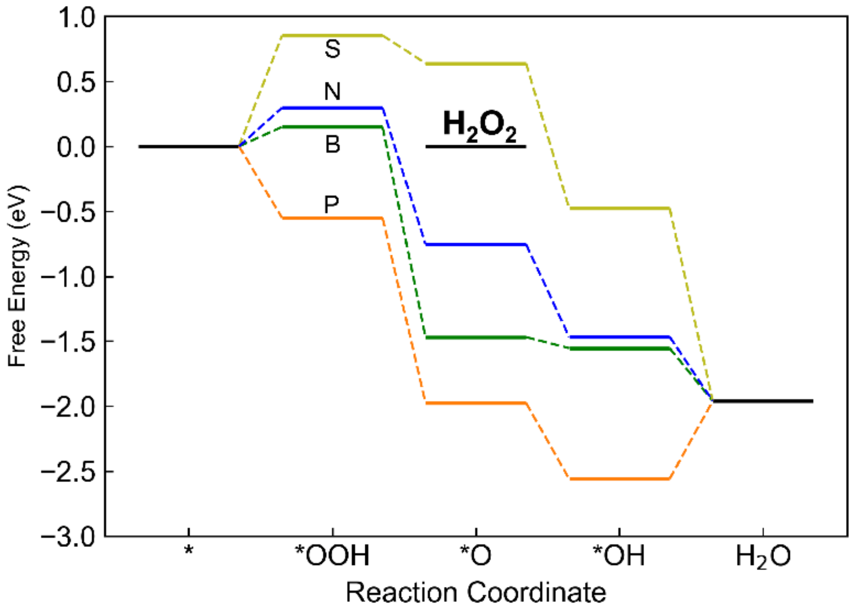

Supplementary Figure 20. Free energy profile of O_2 reduction paths where each state is charge neutral. Note that the trend is the same as the one shown in **Figure 3e** when the charge change for each state is considered, with B-C shows the lowest energy barrier and highest activity. It indicates that, compared to experimental results, calculated overpotential is more accurate if charge change is taken into account in the analysis.

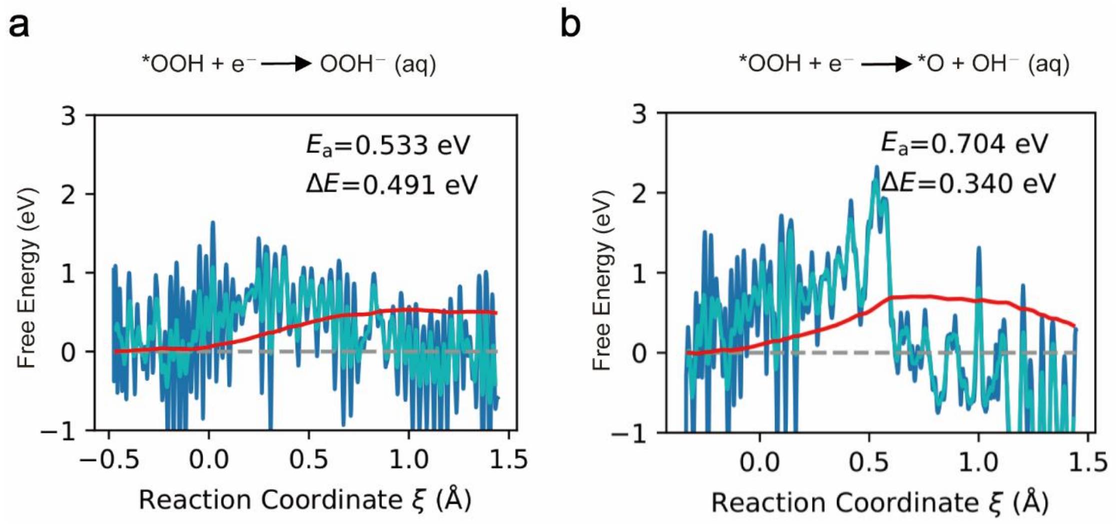

Supplementary Figure 21. Mean force and free energy profile in slow-growth method. (a) $2e^{-}$ pathway **(b)** $4e^{-}$ pathway.

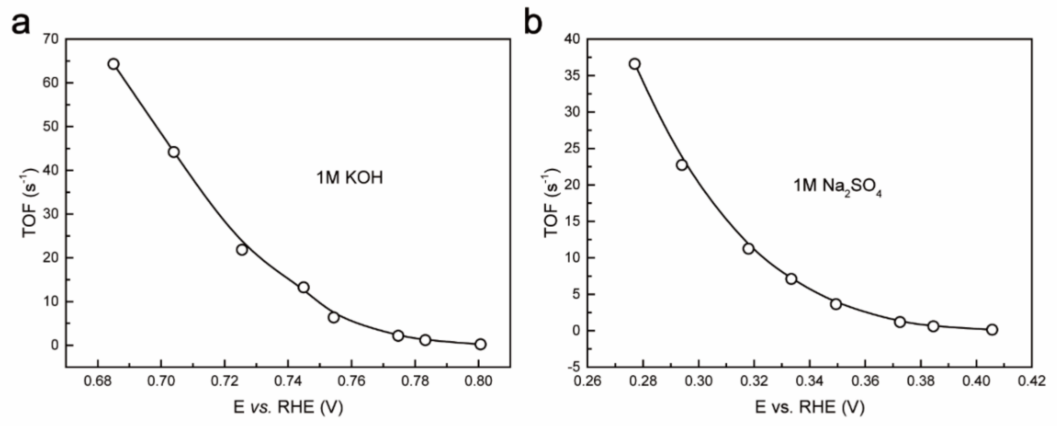

Supplementary Figure 22. Turnover frequency (TOF) normalized by ECSA for B-C in (a) 1M KOH and (b) 1M Na_2SO_4. The ECSA is calculated by electrochemical double layer capacitance (EDLC) measurement illustrated by **Supplementary Figure 24-25** for 1M KOH and 1M Na_2SO_4, respectively.

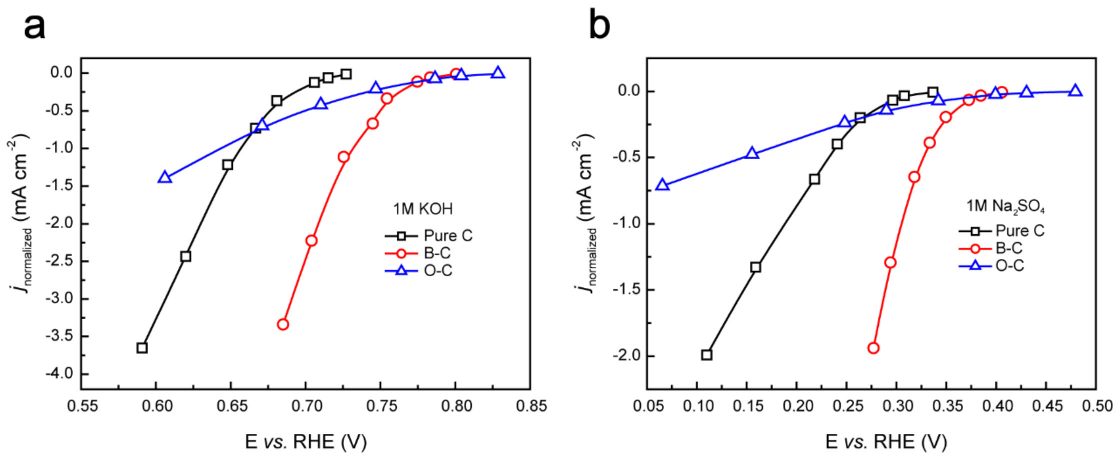

Supplementary Figure 23. I-V curve for three-electrode flow cell test after normalized by electrochemical active surface area (ECSA) (a) in 1M KOH **(b)** in 1M Na_2SO_4. The I-V curves were averaged from 2~3 independent tests for each of the samples. Note that all the I-V curves were manually *iR*-compensated. The B-C still shows the best activity and kinetics after normalized by ECSA.

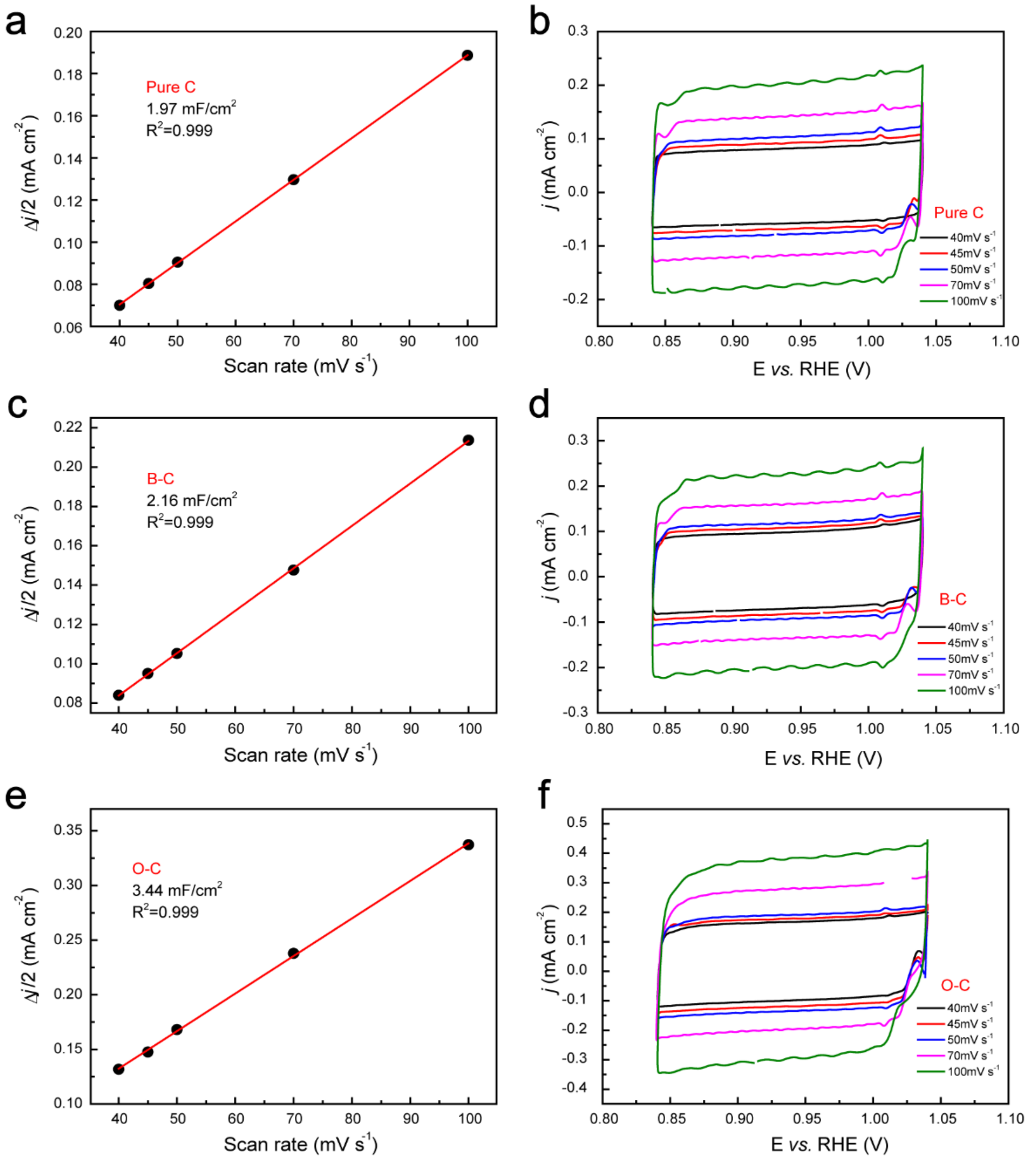

Supplementary Figure 24. Electrochemical double layer capacitance (EDLC) measurement for ECSA of (a-b) Pure C; (c-d) B-C; (e-f) O-C, in 1M KOH.

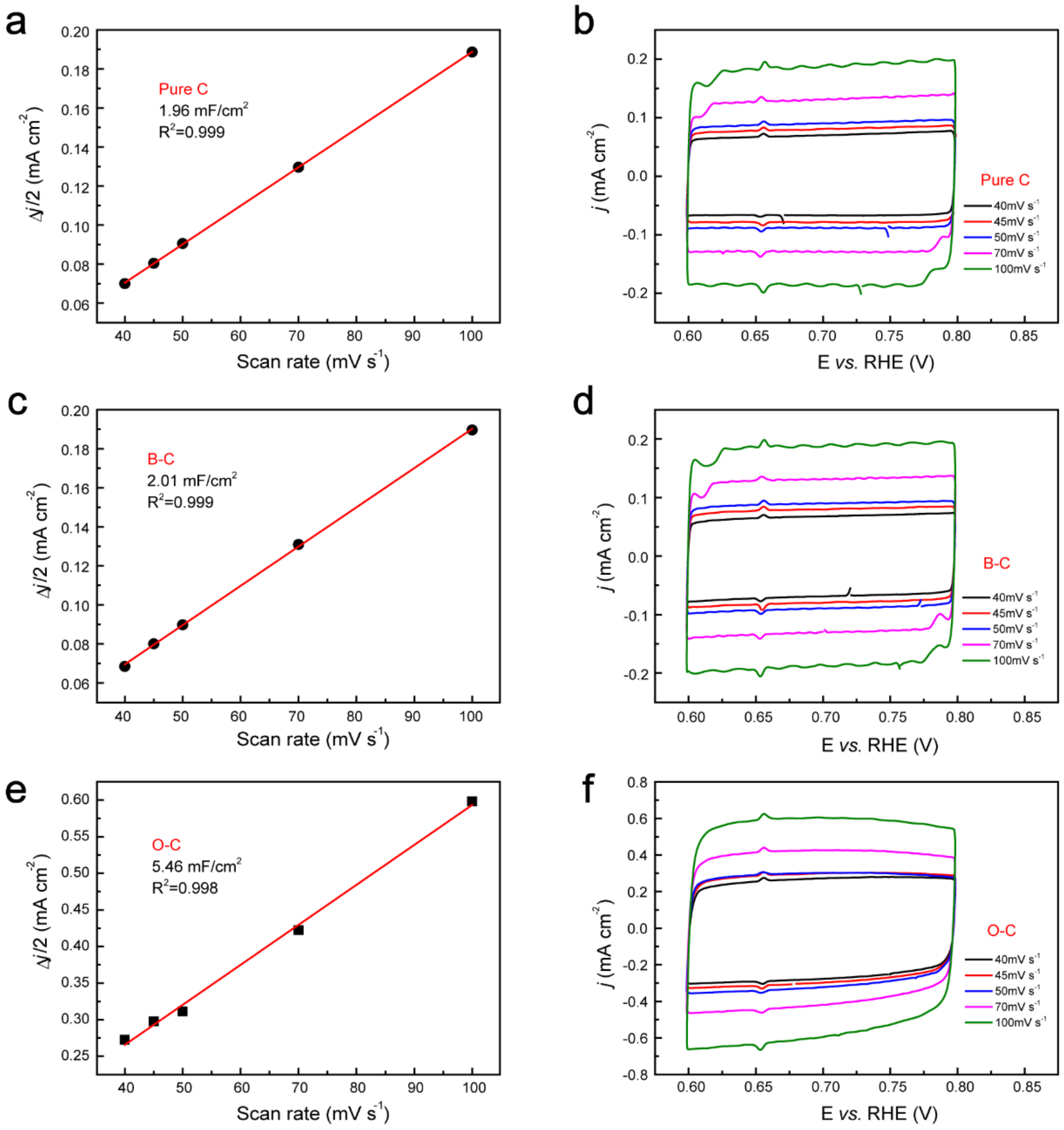

Supplementary Figure 25. Electrochemical double layer capacitance (EDLC) measurement for ECSA of (a)(b) Pure C; (c)(d) B-C; (e)(f) O-C, in 1M Na_2SO_4.

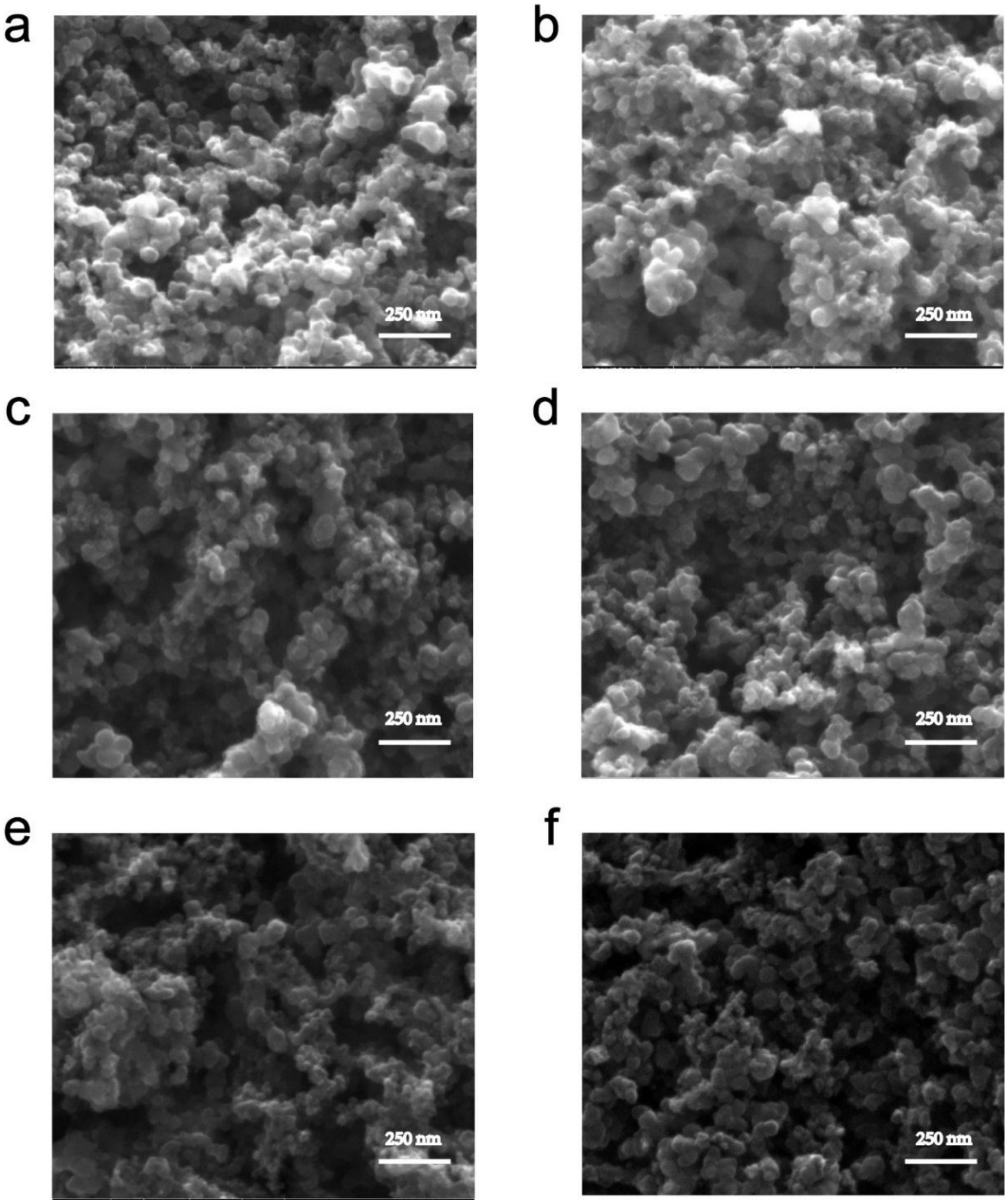

Supplementary Figure 26. SEM morphology images for (a) Pure C **(b)** B-C **(c)** O-C, before performing three-electrode flow cell ORR performance test, and **(d)** Pure C **(e)** B-C **(f)** O-C, after the reaction. The scale bar represents 250 nm. The intact carbon black morphology shown here in SEM of all the samples for both before and after the reaction excludes any morphological effect in the performance in the standard three-electrode flow cell test.

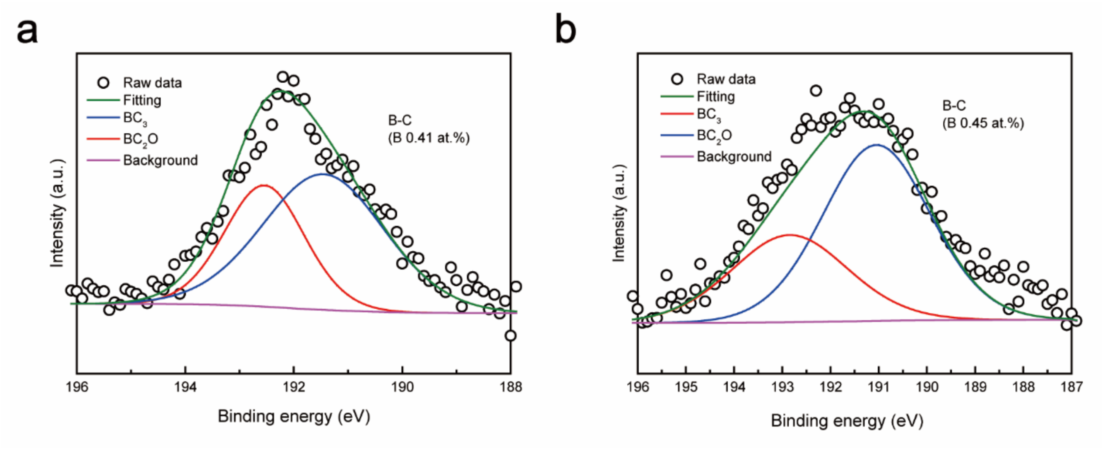

Supplementary Figure 27. XPS High resolution B peak analysis before and after ORR. (a) before ORR (b) after ORR. The oxidation state of boron after the reaction has been well kept, and the boron atomic ratio kept similar, indicating the boron species has not been oxidized during ORR. Note that a.u. represents arbitrary units.

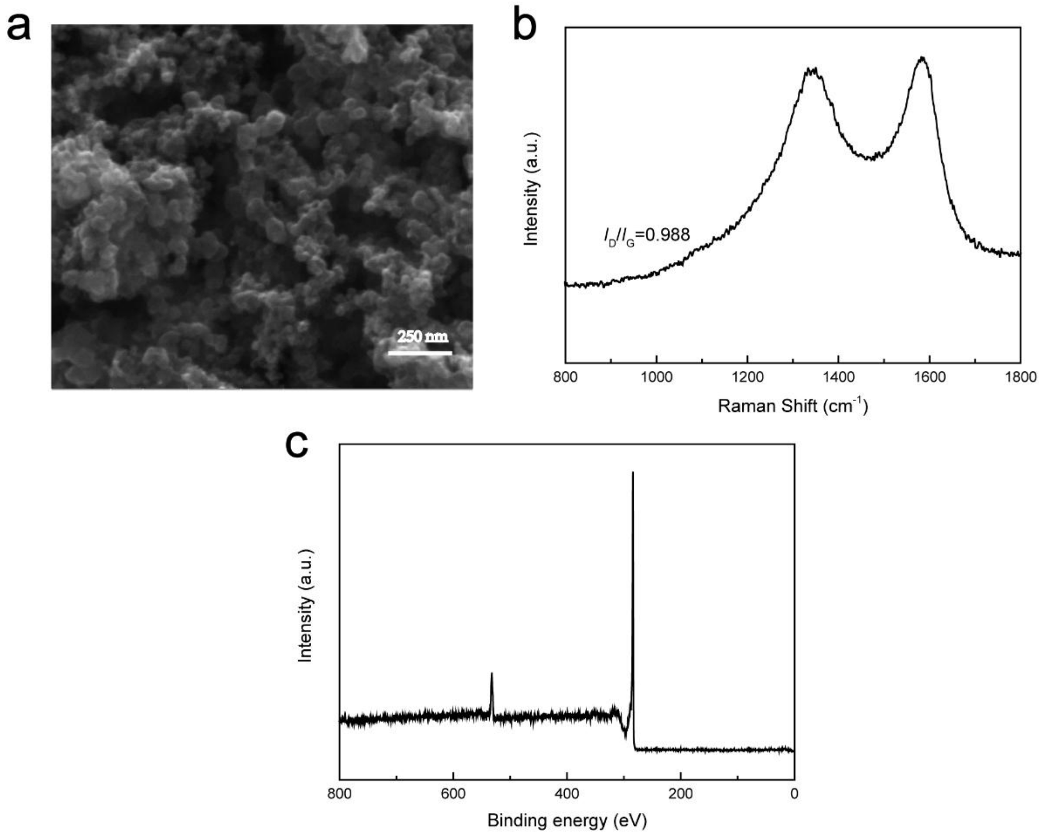

Supplementary Figure 28. Characterization of oxidized carbon sample (O-C). (a) SEM **(b)** Raman. The morphology and defect concentration are similar to those of B-C. **(c)** XPS Survey. Obvious O peak (~532.8 eV) is observed in the survey spectra, indicating successful activation of carbon black. Note that a.u. represents arbitrary units.

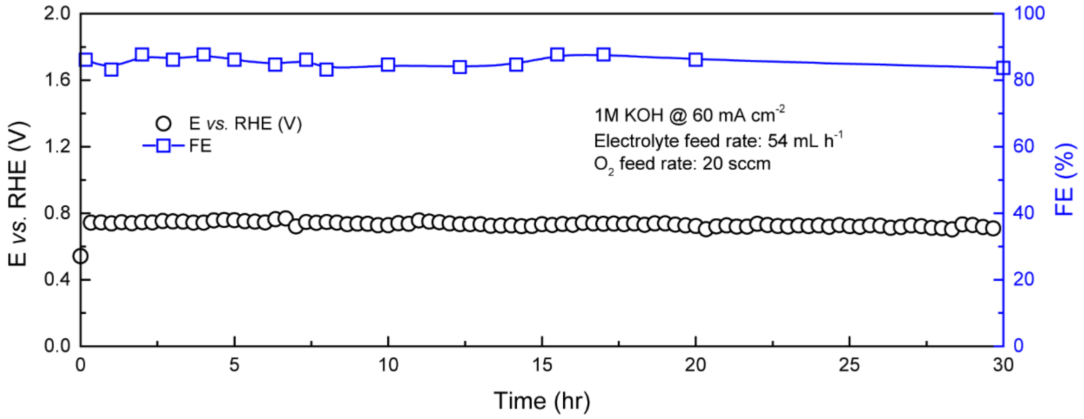

Supplementary Figure 29. Stability performance test for B-C in 1M KOH in three-electrode flow cell configuration. The current is fixed at 60 mA cm^{-2}, with the electrolyte feeding rate fixed at 54mL h^{-1} and oxygen feeding rate fixed at 20 sccm. The catalyst retains its catalytic activity and faradaic efficiency for 30 hours without any degradation, indicating its excellent stability.

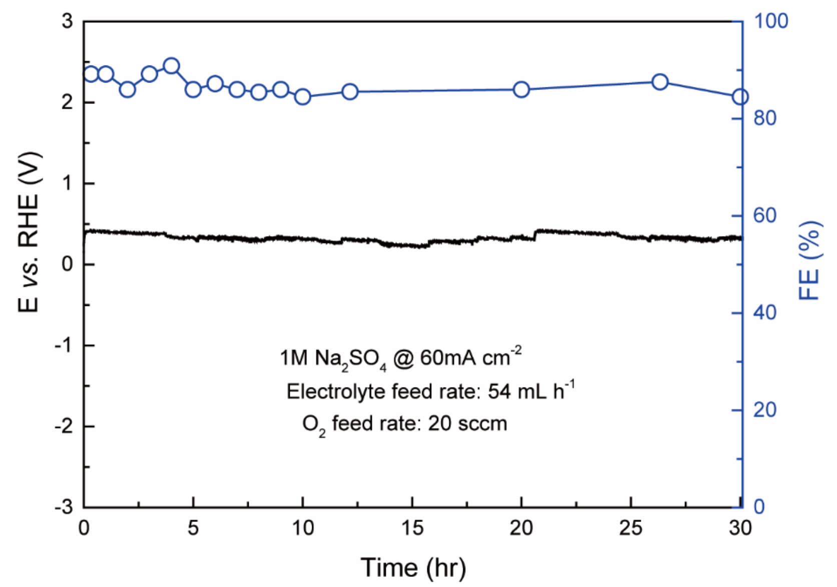

Supplementary Figure 30. Stability performance test for B-C in 1 M Na_2SO_4 in three-electrode flow cell configuration. The current is fixed at 60 mA cm^{-2}, with the electrolyte feeding rate fixed at 54 mL h^{-1} and oxygen feeding rate fixed at 20 sccm. The catalyst retains its catalytic activity and faradaic efficiency for 30 hours without any degradation, indicating its excellent stability.

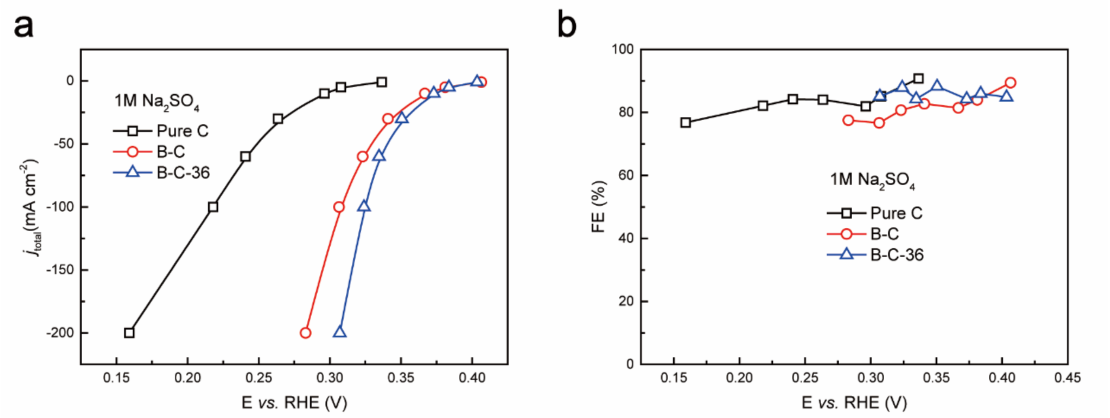

Supplementary Figure 31. Three-electrode flow cell performance of Pure C, B-C and B-C-36 in 1M Na_2SO_4. (a) I-V curve and **(b)** corresponding faradaic efficiencies. Note that all the I-V curves and faradaic efficiency were taken average of 2 independent tests for each of the samples. All the I-V curves are manually *iR*-compensated.

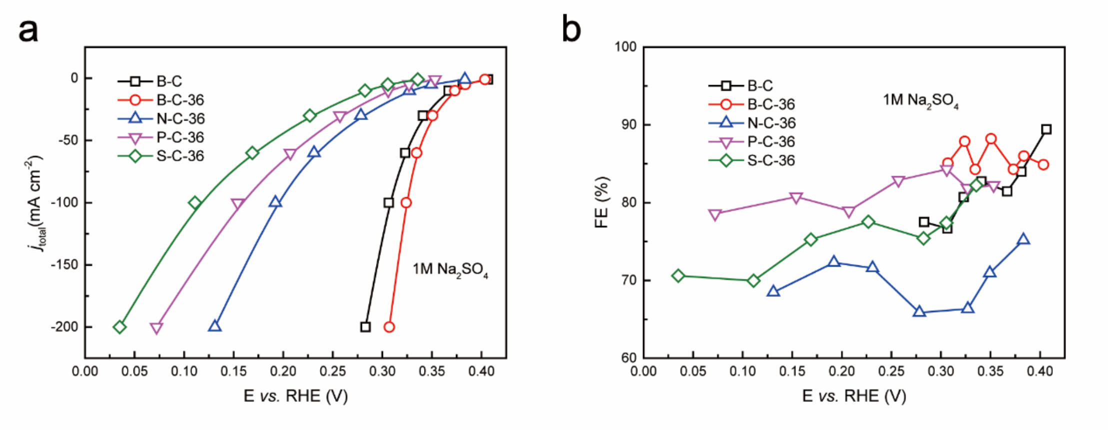

Supplementary Figure 32. Three-electrode flow cell performance of catalysts. (a) I-V curve for B-C, B-C-36, N-C-36, P-C-36 and S-C-36 in 1 M Na_2SO_4 **(b)** Corresponding faradaic efficiencies measured. All the I-V curves are manually *iR*-compensated. B-C-36 shows highest selectivity among all the dopants, with the best activity as well. Furthermore, B-C with lower B loading still shows superior ORR performance compared to other dopants in the X-C-36 (X stands for N, P and S here) series with higher dopant level.

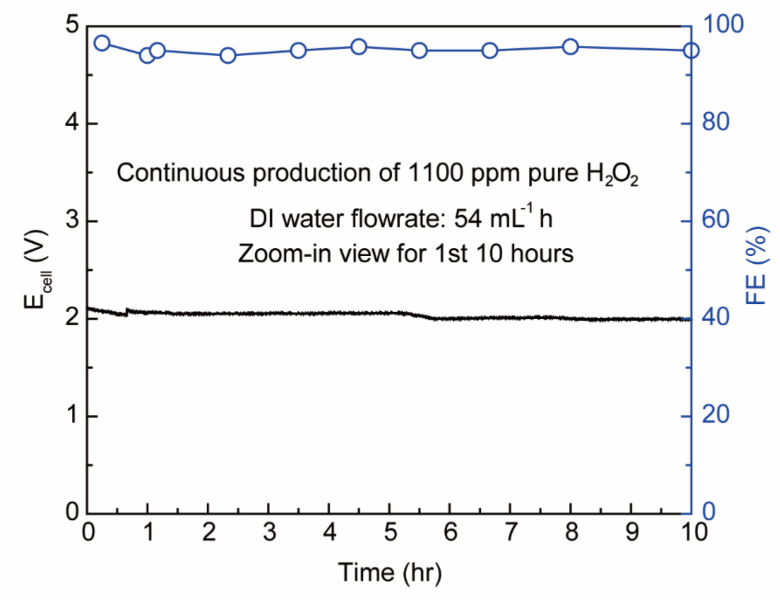

Supplementary Figure 33. Zoom-in view for the 1st 10 hours of long-term pure H_2O_2 production of B-C in solid-electrolyte cell configuration in Figure 6d.

Supplementary Table 1. Surface elemental ratio based on XPS. The values represent atomic ratio (at. %). The trace of surface oxygen remained should come from trace amount of oxygen dopant which is impossible to remove when annealing the samples at the high temperature (750 °C) or due to the oxidation during the sample exposure to air (see more details in **Supplementary Figure 15** and **Supplementary Figure 16**).

	Carbon C	Oxygen O	Doped Element
Pure C	99.01	0.99	N/A
N-C	98.62	0.51	0.86 N
P-C	98.77	0.89	0.34 P
S-C	98.50	0.60	0.90 S
B-C	98.34	1.25	0.41 B

Supplementary Table 2. Electron Probe Micro-Analysis (EPMA) of B-doped carbon

No.	B	C	Total	B	C	B	C	B (D.L.)	C (D.L.)
units	wt. %	wt. %	wt. %	atom	atom	error%	error%	ppm	ppm
1	0.586	99.57	100.155	0.650	99.351	10.84	1.32	540	5638
2	0.538	98.87	99.406	0.600	99.400	12.29	1.33	589	5777
3	0.477	97.77	98.243	0.539	99.461	13.84	1.33	604	5629
4	0.494	100.6	101.047	0.542	99.458	12.61	1.33	555	5441
5	0.609	101.3	101.890	0.664	99.336	10.56	1.33	544	5480
6	0.544	99.84	100.387	0.602	99.398	12.08	1.33	584	5635
Average	0.541	99.647	100.155	0.599	99.401	12.04	1.33	569	5600
St Dev*	0.051	1.239	1.270	0.052	0.052	1.20	0.01	27	122

error%: analytical standard deviation 1σ, based on peak counts and counting times

D.L.: detection limit (in ppm)

St Dev*: compositional range standard deviation 1σ

Supplementary Table 3. Adsorption free energy (in eV) of *OOH on different doping sites. "×" indicates the site is unstable or the chemical adsorption of *OOH is not found. The most active site for H_2O_2 formation (in thermodynamic point of view) are colored in green.

dopant	SV	C-SV	DV	C-DV	5577	C-5577
B	0.152	0.837	×	×	-0.486	-0.269
N	×	0.298	×	×	×	-0.671
P	-1.309	×	-0.551	0.786	-0.901	-0.905
S	×	×	×	0.853	×	×

Supplementary Table 4. Comparison of electrochemical O_2-to-H_2O_2 performance under high currents with state of-the-art catalysts.

Catalyst	Electrolyte	Potential	j_{total} (mA cm^{-2})	FE (%)	Stability
**This work**	**1M KOH**	**0.685 V *vs.* RHE**	**300**	**85.1**	**200 hrs @ 30 mA cm^{-2}**
	**1M Na_2SO_4**	**0.277 V *vs.* RHE**	**300**	**83.2**	
	**DI water (Solid-electrolyte reactor)**	**2.55 V**	**400**	**85.5**	
Fe-O-CNT[4]	1M KOH	0.76 V *vs.* RHE	45	95.4	8 hrs @ 1-3 mA cm^{-2}
Natural air diffusion electrode[5]	0.05 M Na_2SO_4	Not mentioned	240	66.8	20 hrs @ 60mA cm^{-2}
O-CNT[6]	1M KOH	0.68 V *vs.* RHE	40	90	10 hrs @ 0.2-0.4 mA
$Ni-N_2O_2/C$[7]	0.1M KOH	0.5 V *vs.* RHE (without *iR*-compensation)	70	91	8 hrs @ 70 mA cm^{-2}
Co-N-C[8]	0.1M KOH	~0.55 V *vs.* RHE	50	Not mentioned	110 hrs @ ~2.4 mA cm^{-2}
C-PTFE electrode[9]	0.05M Na_2SO_4	Not mentioned	145	29	N/A
$PtP_2-Al_2O_3$[10]	membrane fuel cell (H_2 anode)	Not mentioned	150	78.8	110 hrs @ 0.4V (current density not mention)
N-doped mesoporous carbon[11]	0.1M KOH	0.3 V *vs.* RHE	2 in RRDE	82	6 hrs @ 3 mA cm^{-2}
	0.1M K2SO4	0.2 V *vs.* RHE	3 in RRDE	75	6 hrs @ 3.5 mA cm^{-2}
B,N doped carbon[12]	0.1M KOH	0.55 V *vs.* RHE	1.5 in RRDE	85	50 hrs @~ 1.2 mA cm^{-2}

N-doped carbon nanohorns[13]	0.1M NaOH	0.65 V *vs.* RHE	~0.6 in RRDE	~65	25 hrs @ ~0.6 mA cm^{-2}
	0.1M PBS	0.45 V *vs.* RHE	~0.5 in RRDE	~90	25 hrs @ ~0.5 mA cm^{-2}

Supplementary Table 5. Surface elemental ratio based on XPS for B-C before and after ORR. The values represent atomic ratio (at. %). The B atomic ratio remained similar after the reaction, indicating the doping is stable under the reaction condition.

Atomic Ratio	C (at. %)	B (at. %)	O (at. %)
B-C before reaction	98.34	0.41	1.25
B-C after reaction	98.5	0.45	1.05

Supplementary Table 6. Surface elemental ratio based on XPS for Pure C, B-C and B-C-36. The values represent atomic ratio (at. %).

Atomic Ratio	C (at. %)	B (at. %)	O (at. %)
Pure C	99.01	0	0.99
B-C	98.34	0.41	1.25
B-C-36	98.24	0.91	0.85

Supplementary Table 7. Surface elemental ratio based on XPS for X-C-36 series (X represents B, N, P and S). The values represent atomic ratio (at. %).

Atomic Ratio	C (at. %)	Heteroatom (at. %)	O (at. %)
B-C-36	98.24	0.91	0.85
N-C-36	98.00	1.12	0.88
P-C-36	98.38	0.70	0.92
S-C-36	98.42	1.06	0.52

Supplementary Table 8. Free energy corrections (in eV) for gas phase and surface species

**Species**	*ZPE*	*H-TS*	*Correction*
H_2O_2(g)	0.699	-0.612	0.087
O_2(g)	0.136	-0.543	-0.407
H_2O(g)	0.574	-0.485	0.089
H_2(g)	0.271	-0.319	-0.047
*OOH	0.450	-0.079	0.371
*O	0.072	-0.013	0.059
*OH	0.364	-0.033	0.331

Supplementary Note 1

In the following paragraph, we have performed a simple analysis for energy cost. Please note that the only cost we consider at this stage are energy and feedstock cost (oxygen, electricity, DI water, etc.) but without any equipment cost, i.e. operation cost.

If we are operating the solid-electrolyte cell under highest H_2O_2 production rate condition (see **Figure 6c**): 2.55 V (500 mA, ~0.84 wt.% H_2O_2), with a H_2O_2 production rate of 7.36 mmol cm^{-2} h^{-1} (1.00096 g h^{-1}). The mass of H_2O_2 generated using 1 kWh of electricity will be:

$$m_{H2O2} = \frac{1\ kWh}{2.55\ V * 0.5\ A} * 1.00096\ \mathrm{g\ h^{-1}} = \sim 785.1\ g$$

(a) In our actual test, the flow rate of O_2 feed is 20 sccm (20 standard cubic centimeters per minute), which corresponds to 1200 cm^3 hr^{-1}, or 1.2 L hr^{-1}. **We can use one hour as the time basis for a simple calculation.** Based on the Ideal Gas Law, i.e. PV=nRT, at standard condition (assuming T=273.15K, P=1atm, R=0.0821(L*atm/(mol*K))) with V=1.2L, n=0.0535mol, which gives the mass of O_2 supply to be:

$$mass\ of\ O_2 = 0.0535mol\ of\ O_2\ \times \frac{32g}{mol} = 1.71g\ O_2$$

From our previous calculation, at 2.55 V (500 mA, ~0.84 wt.% H_2O_2), the H_2O_2 production rate for one hour is 7.36 mmol cm^{-2}, i.e. 1.00096 g. Thus, the O_2 cost per mass of H_2O_2 produced is:

$The\ mass\ cost\ of\ O2 = \frac{1.71\ g\ O2}{1.00096\ g\ H2O2} = \frac{1.708g\ O2}{g\ H2O2\ produced} =$ 1.708kg O_2/kg H_2O_2 produced

Given the industrial O_2 price set to be about < $0.1/kg (source: https://www.intratec.us/chemical-markets/oxygen-price), the total cost of O_2 feed can be summed as 0.1708 dollars (17.1 cents) (**assuming *$0.17/kg-H₂O₂* for O₂ price**; but in reality, the cost is even lower). Please note that the O_2 cost can be further reduced by collecting and recycling O_2 gas produced from OER on the anode side.

(b) Assuming the price of electricity is 3 cents/kWh[14], we can roughly estimate a electricity for H_2O_2 production to be ***$0.038/kg-H₂O₂***.

(c) Since we locate in Texas, US, we just use the water price in Texas as our calculation basis. The industrial water in Texas is about $1.91/(1000 gallon), or $0.00191/gallon (https://www.fbgtx.org/673/Industrial-Water-Rates). The price for deionize one gallon of water is lower than $0.03 (https://blog.uswatersystems.com/2012/08/de-ionization-101/). Thus, the total cost for DI water is less than about $0.03/gallon, i.e. **$0.008/kg**, or about ***$0.9/kg-H₂O₂***.

In summary, the total energy and feed stock cost is less than about ***$1.1/kg-H₂O₂***. Since the largest portion of the cost is from deionizing water, the cost can be further reduced in the future by replacing DI water feed stock with industrial water plus water filter. Furthermore, our on-site generation method does not need the cost for transportation and storage. In comparison, the traditional industrial anthraquinone process for H_2O_2 production has a rough cost of ***$1.5/kg-H₂O₂*** without transportation

and storage cost (*http://www.h2o2.com/faqs/FaqDetail.aspx?fId=25)*. Thus, our method is still far more economical than the traditional way.

Supplementary References

1 Zhang, C., Sunarso, J. & Liu, S. Designing CO 2-resistant oxygen-selective mixed ionic–electronic conducting membranes: guidelines, recent advances, and forward directions. *Chemical Society Reviews* **46**, 2941-3005 (2017).

2 Ouyang, C. *et al.* Boosting the ORR performance of modified carbon black via C–O bonds. *Chemical science* **10**, 2118-2123 (2019).

3 Ma, R. *et al.* A review of oxygen reduction mechanisms for metal-free carbon-based electrocatalysts. *npj Computational Materials* **5**, 1-15 (2019).

4 Jiang, K. *et al.* Highly selective oxygen reduction to hydrogen peroxide on transition metal single atom coordination. *Nature communications* **10**, 1-11 (2019).

5 Zhang, Q. *et al.* Highly efficient electrosynthesis of hydrogen peroxide on a superhydrophobic three-phase interface by natural air diffusion. *Nature communications* **11**, 1-11 (2020).

6 Lu, Z. *et al.* High-efficiency oxygen reduction to hydrogen peroxide catalysed by oxidized carbon materials. *Nature Catalysis* **1**, 156-162 (2018).

7 Wang, Y. *et al.* High-Efficiency Oxygen Reduction to Hydrogen Peroxide Catalyzed by Nickel Single-Atom Catalysts with Tetradentate N2O2 Coordination in a Three-Phase Flow Cell. *Angewandte Chemie International Edition* (2020).

8 Jung, E. *et al.* Atomic-level tuning of Co–N–C catalyst for high-performance electrochemical H 2 O 2 production. *Nature Materials* **19**, 436-442 (2020).

9 Brillas, E., Calpe, J. C. & Casado, J. Mineralization of 2, 4-D by advanced electrochemical oxidation processes. *Water Research* **34**, 2253-2262 (2000).

10 Li, H. *et al.* Scalable neutral H 2 O 2 electrosynthesis by platinum diphosphide nanocrystals by regulating oxygen reduction reaction pathways. *Nature communications* **11**, 1-12 (2020).

11 Sun, Y. *et al.* Efficient Electrochemical Hydrogen Peroxide Production from Molecular Oxygen on Nitrogen-Doped Mesoporous Carbon Catalysts. *ACS Catalysis* **8**, 2844-2856, doi:10.1021/acscatal.7b03464 (2018).

12 Chen, S. *et al.* Designing boron nitride islands in carbon materials for efficient electrochemical synthesis of hydrogen peroxide. *Journal of the American Chemical Society* **140**, 7851-7859 (2018).

13 Iglesias, D. *et al.* N-doped graphitized carbon nanohorns as a forefront electrocatalyst in highly selective O2 reduction to H2O2. *Chem* **4**, 106-123 (2018).

14 Chu, S., Cui, Y. & Liu, N. The path towards sustainable energy. *Nature materials* **16**, 16 (2017).
